# A Comprehensive Investigation on Development of Lightweight Aluminium Miniature Gears by Thermoelectric Erosion Machining Process

**DOI:** 10.3390/mi12101230

**Published:** 2021-10-09

**Authors:** Sujeet Kumar Chaubey, Neelesh Kumar Jain, Kapil Gupta

**Affiliations:** 1 Department of Mechanical and Industrial Engineering Technology, University of Johannesburg, Johannesburg 2028, South Africa; sujeetiiti@gmail.com; 2 Discipline of Mechanical Engineering, Indian Institute of Technology Indore, Indore 453 552, India; nkjain@iiti.ac.in

**Keywords:** thermoelectric erosion, microgeometry, micromanufacturing, miniature, spur gear, surface roughness

## Abstract

Nowadays, size, weight, and durability are crucial factors in product development that draw the attention of many researchers and engineers towards research and innovation in the micromanufacturing area. This paper reports on the development of a lightweight aluminium gear of miniature size with a bore and hub using wire-assisted thermoelectric erosion machining (WTEM). The external spur gear was cut from 7075 aluminium alloy round stepped gear blank by WTEM using 0.25 mm brass wire. Further, the miniature gear was tested for various manufacturing quality parameters such as microgeometry, surface roughness, and microstructure, along with evaluating process productivity in terms of volumetric gear cutting speed To understand the mechanism of development of aluminium miniature gear, an investigation on the influence of WTEM parameters namely servo-voltage, pulse-on time, pulse-off time, and wire speed on surface roughness was conducted. A total of 18 gears were fabricated following Taguchi L_9_ (3^4^) orthogonal array approach of design of experiments considering the randomization and replication. A typical average surface roughness value of 1.58 μm and manufacturing quality of DIN standard number 7 based on gear microgeometry were successfully achieved. Microscopic investigation revealed uniform and accurate tooth profiles, flank surfaces free from burrs and contaminants, and uniform microstructure that confirm the good performance characteristics of the developed lightweight miniature gear of aluminium. This investigation will add new results in the field as regards the development of lightweight microparts.

## 1. Introduction

Globally, the demand for cost-effective and lightweight materials is increasing very rapidly in many industries such as micro-systems, bio-medical, aerospace and electronics to reduce their products costs and weight as well as reduction in their overall energy consumption without compromising their operating performance and service life. It has compelled the exploration of lightweight materials and innovation of their manufacturing and processing to fulfil the needs of modern manufacturing industries. As a result, lightweight materials such as aluminium, magnesium, titanium and their alloys are extensively explored as alternative materials to manufacture micro-parts and components. Miniature gears are one of the important microparts which have widespread application areas from aerospace to biomedical industries [1,2]. Miniature gears of light materials such as aluminium, brass, bronze, copper, and polymers possess specific characteristics, i.e., light in weight, compact size, good performance and low operating power requirement [3,4]. Their manufacturing quality, especially microgeometry and surface finish, plays a crucial role in this [5]. It mainly depends on the selection of gear materials and the type of manufacturing process. Primarily, the selection of materials and manufacturing processes for a gear mainly depends on the application requirements, cost, and types of gear (i.e., cylindrical, conical, and non-circular gears), and the possible operating conditions. Aluminium is a good candidate material for miniature gears [3]. 7075 aluminium alloy has certain characteristics such as lightweight, good strength-to-wear-ratio, good machinability, cost-effectiveness, higher thermal conductivity, higher sound and shock absorption ability, non-magnetic nature, and corrosion resistance [6]. Miniature gears made of aluminium are usually used for high-speed and low-to-medium power transmission [4]. These gears reduce weight and cost and are comprehensively used in several fields such as automotive, food processing, chemical plants, miniaturized gear-pump, gearbox, gear-reducer, actuators, toys, and in several domestic appliances [3,7].

Hobbing and milling are widely used conventional processes for manufacturing miniature size gears. However, certain individual inherent limitations related to materials, shape, size, tool marks on their flank surfaces, non-uniform tooth profile, poor edge definition and manufacturing quality of gears, restrict their applications for manufacturing these gears. Further, improvement in the quality of gears made by these conventional processes needs the assistance of grinding, honing, lapping, and shaving type of finishing processes, which elongates the process chain and adds extra cost. This challenge is being addressed by the researchers and new processes are being exploited [3,8]. 

Wire-assisted thermoelectric erosion machining (WTEM) process is one of the processes which has the potential to possibly generate precision parts of miniature size. It has been used for the manufacturing of microparts including gears because it is a material saving process, requires minimum attention during manufacturing, and is easy to operate and handle [9,10]. Previous work on miniature gear manufacturing by WTEM is limited to brass and stainless-steel materials [11,12,13,14]. To the best knowledge of the authors, attempts on manufacturing lightweight aluminium gears with bore and hub by WTEM process are scarce and a detailed investigation is required to be conducted to complement miniaturization. Therefore, the major objectives of this study are as follows:

- To manufacture lightweight miniature size gears of aluminium by WTEM process.
- To explore the potential of WTEM to generate good microgeometry and surface quality in gears.
- To study variation in surface roughness with parameters of WTEM in order to understand the mechanism of WTEM gear manufacturing.
- To improve the productivity of WTEM process for manufacturing of bored miniature gear with the hub.
- To do modelling of WTEM parameters for future prediction of gear surface quality and microgeometry.
- To examine and evaluate the microgeometry and surface quality of lightweight aluminium miniature gears.

## 2. Experimentation

### 2.1. Material and Specifications

**Figure 1:**
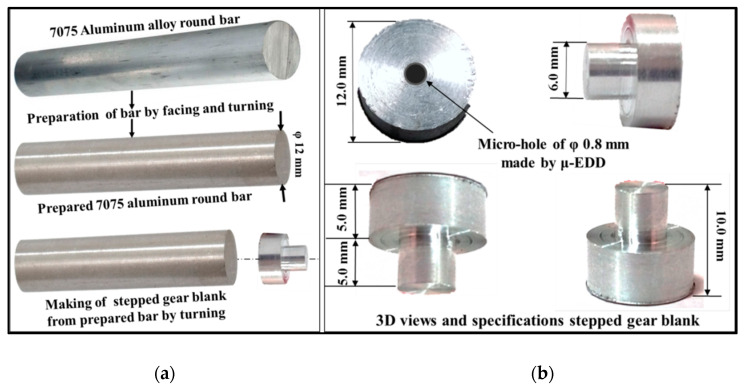

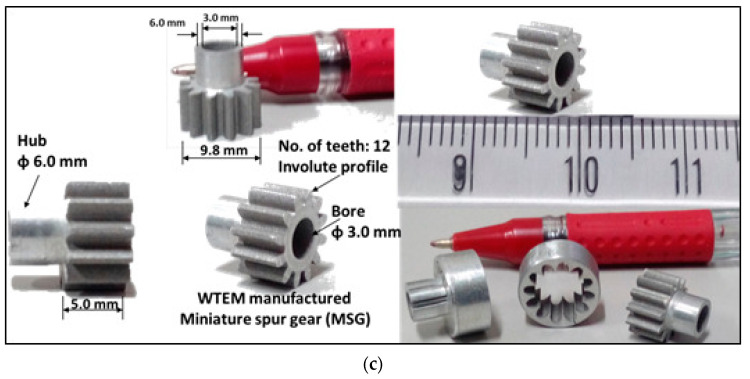
Procedure and specifications of: (**a**) 7075 aluminium alloy bar; (**b**) stepped gear blank; and (**c**) WTEM manufactured miniature spur gear with bore and hub.

In this study, external miniature spur gears having specifications mentioned in Table 1 were manufactured from a stepped blank made from 7075 aluminium alloy bar using a computer numerical controlled (CNC) Sprintcut-Win WTEM machine from Electronica India Limited Pune, India, by a 0.25 mm diameter soft plain brass wire having a tensile strength in the range of 470–510 N/mm^2^. Deionized water at flushing pressure of 7 kg/cm^2^ was used as the dielectric to continuously flush away the debris from the inter-electrode gap (IEG). Manufacturing capabilities of this machine include: 0.8 μm surface finish; 170 mm^2^/min cutting speed; 50 mm face width and ±30° as maximum inclination angle. The gear blank is firmly held in the V block which is mounted on the main work-table with help of clamps. The WTEM main worktable moves along X and Y directions in steps of 1 micron according to the CNC part program for machining of gear teeth. A continuously travelling brass wire fed from the feed spool is passing through the upper guide, gear blank and lower guide and collected on the take-off spool box. During machining, brass wire is always kept in tension between upper and lower guides to avoid wire vibration and ensure the accurate cutting of gear tooth profile. 7075 aluminium alloy is the gear material. The chemical composition of 7075 aluminium alloy used in this work is given in Table 1. 7075 aluminium alloy bars of 200 mm length and 12 mm diameter were prepared by turning and finishing processes on CNC lathe to achieve perfectly round and burr-free gear blanks. Figure 1a–c depicts 3-dimensional views and specifications of the aluminium alloy bar, gear blank and WTEM manufactured miniature size gear with bore and hub respectively.

### 2.2. Procedure of Experiments

Based upon the selection of four WTEM parameters (i.e., servo-voltage ‘*S_V_*’, pulse-on time ‘*T_on_*’, wire speed ‘*W_F_*’, and wire feed ‘*W_F_*’) at three levels each (see Table 1), Taguchi’s L_9_ (3^4^) orthogonal array design approach of design of experiments (DoE) was employed. A total of 9 experiments with 2 replicates each were conducted through which a total of 18 miniature gears of aluminium were fabricated. The selection of WTEM parameters and their levels and values are based on machine constraints, literature review, and trial experiments. 

Volumetric gear cutting speed ‘*VGCS*’ is a measure of the productivity of the WTEM process. VGCS is defined as the volume of material removed during the process of gear cutting to the total time taken for cutting miniature-sized gear and expressed by Equation (1). Total material lost during machining can be expressed by Equation (2).

(1) $$VGCS=\frac{\mathrm{Weight}\;\mathrm{of}\;\mathrm{material}\;\mathrm{loss}\;\left(W_{L}\right)\;\mathrm{during}\;\mathrm{gear}\;\mathrm{cutting}}{\mathrm{Gear}\;\mathrm{material}\;\mathrm{density}\;\left(\rho \right)\times \mathrm{Total}\;\mathrm{gear}\;\mathrm{cutting}\;\mathrm{time}\;\left(t\right)}\;\left(\frac{{\mathrm{mm}}^{3}}{\min.}\right)\;$$

(2) $$\mathrm{Where},\;\;W_{L}=W_{before}-(W_{after\;}+W_{bar}+W_{gear})\;\left(g\right)$$

Here, *W_before_*_,_ and *W_after_* are the weight of the gear blank before and after machining respectively, while *W_gear_* and *W_bar_* are the weight of the gear and weight of bore bar obtained after enlarging micro-hole, respectively. *ρ* stands for a density of gear materials and *t* stands for total cutting time per gear. Weight of gear blank, gear, and bore bar were measured on precision weighing machine (Model: DS 852G from Essae group company, India) having accuracy up to 0.01 g. The manufacturing time of miniature gear was directly noted from the WTEM machine tool display unit.

The micro-level unevenness on the gear tooth flank surfaces is determined by surface roughness parameters. A higher value of surface roughness causes early failure due to excessive wear and friction on flank surfaces of a gear [15]. Surface roughness has several parameters, but average surface roughness ‘*R_a_*’ and the total height of roughness profile ‘*Rt*’, also known as maximum surface roughness, are the two most important parameters referred to in the manufacturing of engineered microparts. These parameters significantly affect the service life, noiseless and smooth operating performance of a gear [14,15]. 

A deviation in microgeometry parameters of gear determines its manufacturing quality [3,4,5]. For that, the two most important categories are form errors and location errors that significantly affect the noise behaviour and accuracy of motion transfer of a gear. Total profile error ‘*F_a_*’ and total pitch error ‘*F_p_*’ are important factors of a form error and location error of a gear, which affect noise characteristic and load carrying ability of a gear respectively. Topography evaluation provides complete detail of gear tooth flank surface peaks and valleys. This section may be divided into subheadings. It should provide a concise and precise description of the experimental results, their interpretation, as well as the experimental conclusions that can be drawn.

### 2.3. Procedure for Manufacturing of Miniature Spur Gear of Aluminium

**Figure 2:**
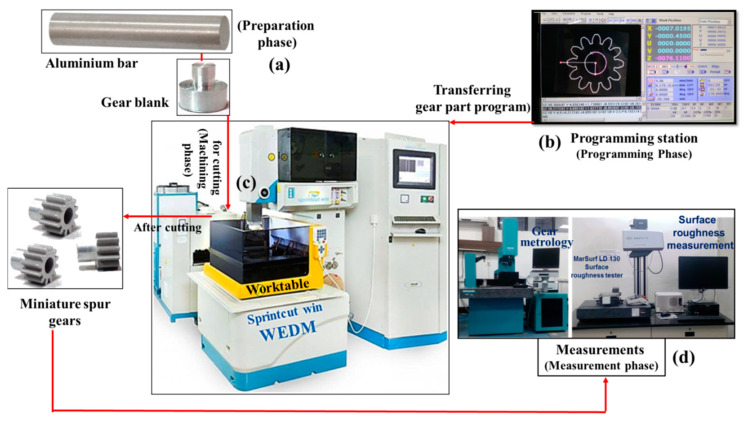
Process sequence or different phases involved to manufacture miniature spur gear with bore and hub by wire-assisted thermoelectric erosion machining: (**a**) preparation phase; (**b**) programming phase; (**c**) machining phase; and (**d**) measurement phase.

Following are the phases involved in developing lightweight miniature gear of aluminium by wire-assisted thermoelectric erosion machining (see Figure 2): 

#### 2.3.1. Preparation Phase

**Figure 3:**
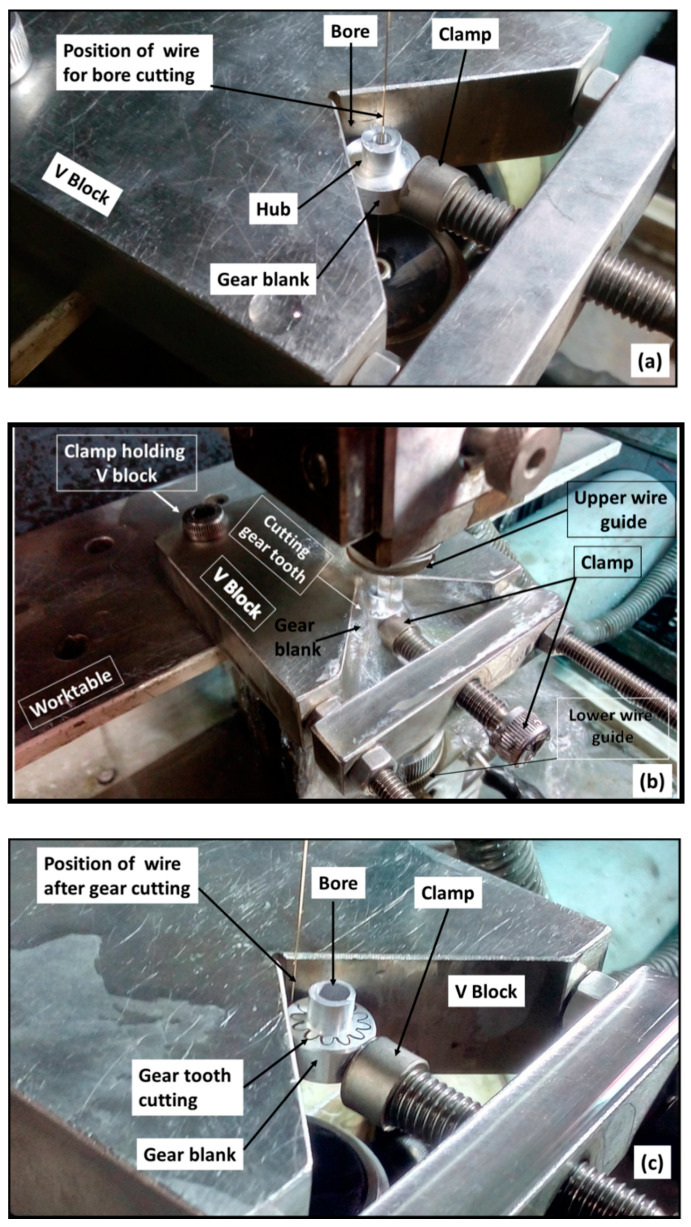
Illustration of different steps involved during cutting of miniature spur gear with bore by WTEM: (**a**) clamping of V block on WTEM worktable and holding gear blank in the V block through the clamp and inserting and positioning wire at the centre of micro-hole for bore cutting; (**b**); cutting of gear teeth; (**c**) positioning of wire after cutting a gear.

- Preparation of the 7075 aluminium alloy bar on CNC lathe.
- Manufacturing of precise stepped gear blanks on CNC lathe to ensure perfect clamping and positioning of gear blank in the V block with respect to the wire on CNC WTEM machine as illustrated in Figure 3.

- Micro-hole (800 μm diameter) drilling at the centre of the prepared stepped gear blank. The micro-hole acts as an entry path to the brass wire of 250 μm diameter for manufacturing of bore of miniature gear.

#### 2.3.2. Programming Phase

**Figure 4:**
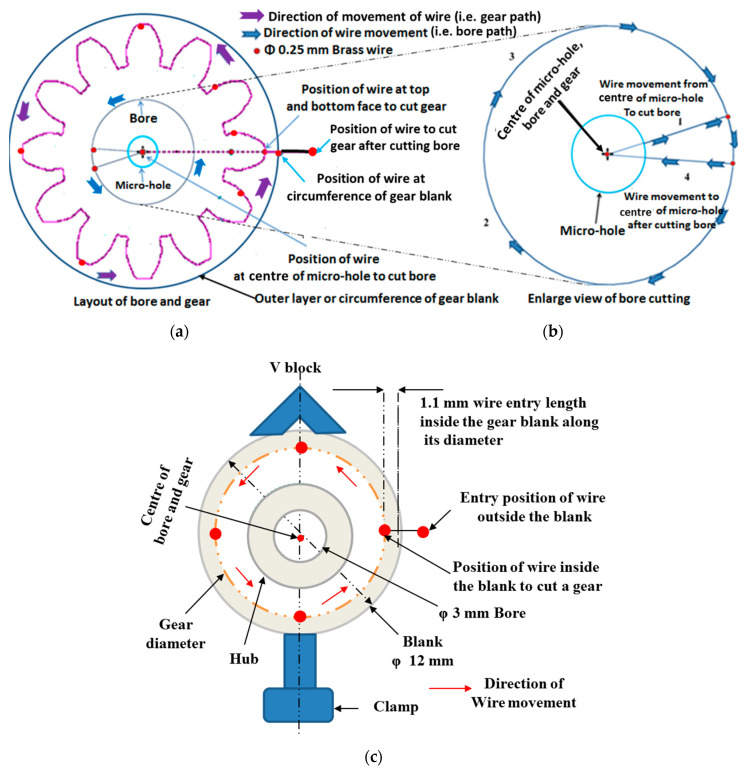
Layout of miniature gear with bore and direction of the wire movements during cutting of bore and gear by WTEM process: (**a**) gear and bore; (**b**) enlarge view of bore along with the path of wire movement, and (**c**) schematic diagram of gear blank, V block, clamp, and wire entry position for cutting miniature gear.

- The next step is preparing a layout of the miniature size gear with the bore in the ELCAM software as depicted in Figure 4. Then using this geometric information to make a combined part program for bore diameter and gear as per their specification by window-based part programming ELCAM software incorporated with a workstation of CNC WTEM machine.
- Transferring the part program to the WTEM machine through data exchange cable, floppy disc or pen-drive.

#### 2.3.3. Machining Phase

- The first step of the machining phase is clamping of the V block in a perfectly horizontal position on the worktable of the CNC WTEM machine with the help of a dial gauge.
- Ensure firm and appropriate positioning of the prepared stepped gear blank with respect to the brass wire in the V block with the help of clamp as shown in Figure 3. Even minute variation in perfect positioning of V block on the worktable and the prepared gear blank in V block may cause inaccurate positioning of gear blank with respect to the brass wire that leads to inaccurate or taper bore cutting and thus significantly deteriorate the quality of gear tooth profiles.
- The next step of machining is inserting the wire through the micro-hole and then locating it at the centre of the micro-hole as shown in Figure 3b. Then enlarging it up to 3 mm diameter to make it bore by WTEM process as depicted in Figure 3c and Figure 4.
- After enlarging the micro-hole, the next step is breaking the brass wire and moving the wire to position it at the entry point of gear which lies between gear blank and V block by running the WTEM machine in dry mode condition as shown in Figure 2c and Figure 4. In dry mode condition, no sparking and flushing take place during machining.
- Cutting of tooth profile of miniature spur gear by moving the wire in a pre-defined path according to the part program of gear as shown in Figure 3a–c and Figure 4.

#### 2.3.4. Measurement Phase

- The next step after gear cutting is measurements of volumetric gear cutting speed and surface roughness (Figure 2) of WTEM manufactured miniature spur gear with bore and hub.
- The next step is microgeometry measurements and microstructure examination of the best finish gear made by WTEM. This section may be divided into subheadings. It should provide a concise and precise description of the experimental results, their interpretation, as well as the experimental conclusions that can be drawn.

### 2.4. Measurement

Mahr metrology makes LD 130 tester was used to measure the average surface roughness *‘Ra’* and the total height of roughness profile *‘Rt’*. Surface roughness measurements were performed by tracing a 5 µm tip diameter probe along the profile or in the direction of wire path on both left and right-hand tooth flank surfaces of two radially opposite tooth of the gear. Measurements were taken by considering cutoff length 0.08 mm; evaluation length 1.0 mm and Gaussian filter to differentiate roughness and waviness profile and average roughness value of both tooth flanks was considered for evaluation. Precision weighing machine (Model: DS 852G from Essae group company, India) having accuracy up to 0.01 g was used to measure the weight of gear blank, bore bar and machined gear to calculate the total material loss during manufacturing of this miniature gear by WTEM. Total material loss during gear cutting is useful to determine the productivity of the WTEM process while a digital stopwatch having a least count of 0.01 s was used to determine the time taken for manufacturing miniature gear. Total profile error ‘*F_a_*’ and total pitch error ‘*F_p_*’ were measured on CNC gear metrology machine (Model: SmartGEAR 500; Made: Wenzel GearTech, Germany). German standard DIN 3962 was referred for manufacturing quality assignment. It is important to mention that DIN ranges from 1 (best quality) to 12 (lowest quality). Microscopic investigation of the tooth profile and bore of the best finish miniature spur gear made by WTEM was done on Carl Zeiss make SUPRA 55 scanning electron microscope (SEM).

## 3. Results and Discussion

**Figure A1:**
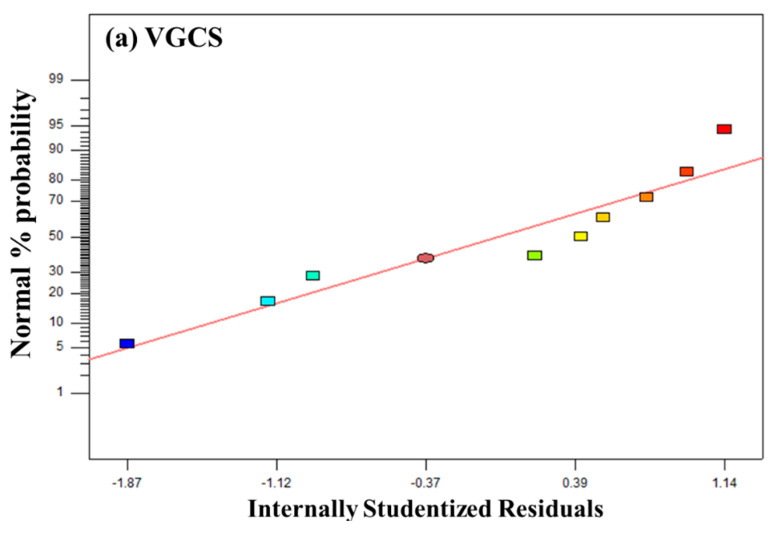

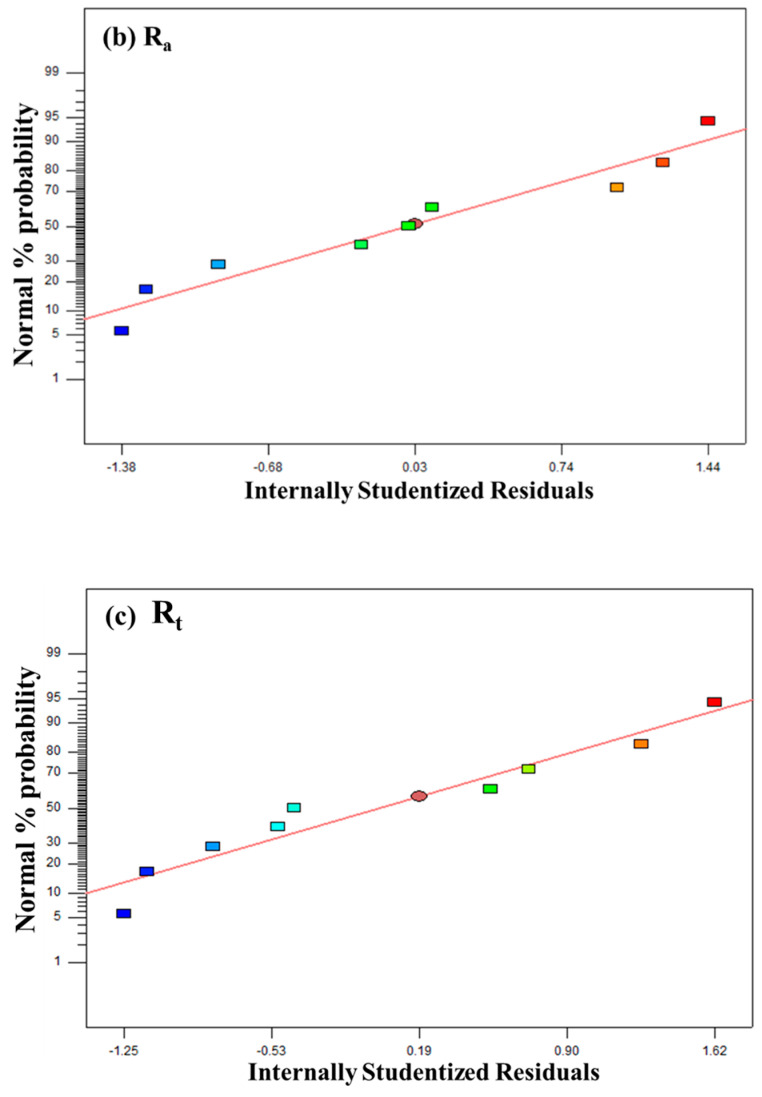
Normal graphs of residuals for considered responses: (**a**) volumetric gear cutting speed ‘VGCS’; (**b**) average surface roughness ‘*R_a_*’, and (**c**) maximum surface roughness ‘*R_t_*’.

Table A1 presents the results for all nine experimental runs. The residual analysis was used as the primary diagnostic tool and the graphs of normal probability plot of residuals have been drawn for the responses and presented in Figure A1. It can be observed from these graphs that most residuals are accumulated around a straight line and normally distributed. Modelling equations for considered responses were developed by regression analysis using experimental values and analysis of variance (ANOVA) using 95% confidence interval was studied to identify the significance of the developed regression models and the significant variable parameters affecting the finish of the tooth flank surfaces and WTEM productivity for miniature gear manufacturing. Results of regression analysis and ANOVA are presented in Table A2 and found that (i) servo-voltage, pulse-on time, wire speed are significantly affecting gear cutting speed and *average* surface roughness ‘*R_a_*’*,* while for the total height of roughness profile parameters ‘*R_t_*’, servo-voltage, pulse-on time and wire tension are significant, (ii) the developed regression models are found to be significant at 95% confidence interval. The developed regression equations to predict the values of responses are as follows:

(3) $$VGCS=-6.64+0.0997\;S_{V}+12.2\;T_{on}+0.240\;W_{S}\;\;\;\;\;\;\;\;\left(\frac{{\mathrm{mm}}^{3}}{\min.}\right)$$

(4) $$R_{a}=1.36+0.00700\;S_{V}+0.408T_{on}-0.0183\;W_{S}\;\;\;\;\;\;\;\;\;\;\;\;\left(\mu m\right)$$

(5) $$R_{t\;}=5.87+0.148\;S_{V}+4.69T_{on}-0.00131\;W_{T}\;\;\;\;\;\;\;\;\;\;\;\;\;\;\;\left(\mu m\right)$$

It was observed from this study that signal to noise ratio and multi-response optimization techniques such as desirability function analysis (DFA) cannot be performed for experimental data obtained from Taguchi L_9_ (3^4^) due to the zero values of error and denominator of the F-test.

**Figure 5:**
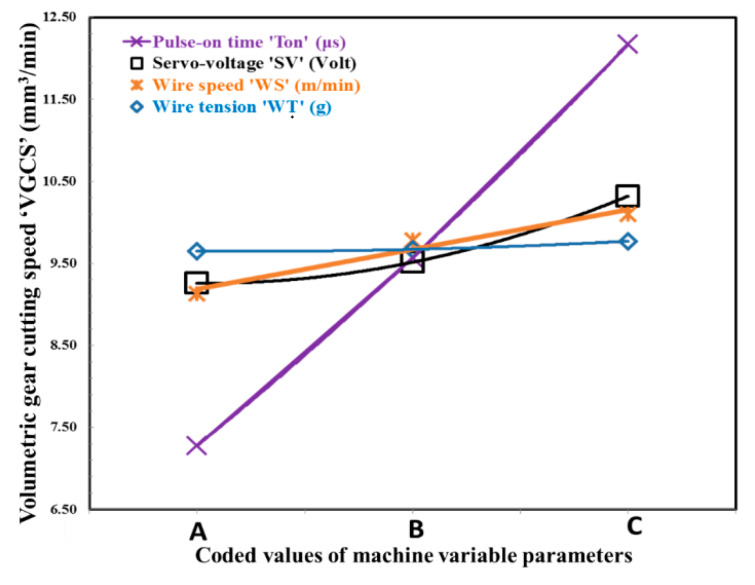
Coded values of machine variable parameters and their effect on volumetric gear cutting speed.

**Figure 6:**
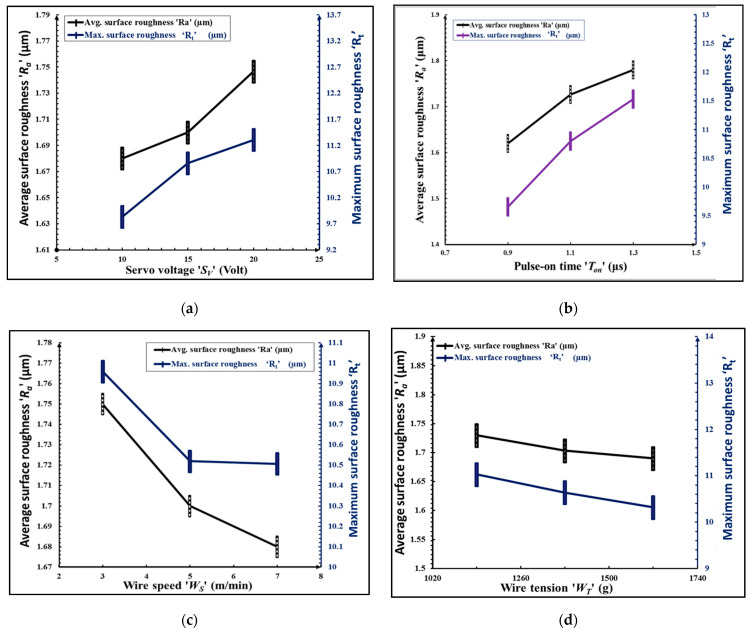
Influence of WTEM parameters on average surface roughness ‘*R_a_*’ and the total height of roughness profile ‘Rt’ measured at tooth flanks of miniature gear: (**a**) servo-voltage ‘*S_V_*’; (**b**) pulse-on time ‘*T_on_*’; (**c**) wire speed ‘*W_S_*’; and (**d**) wire tension ‘*W_T_*’.

Figure 5 and Figure 6 graphically represent the variations of *VGCS*, *R_a_*, and *R_t_* with WTEM parameters. It can be observed that to maximize *VGCS*, there exist optimum ranges of servo-voltage. It can be seen from Figure 5 that (i) *VGCS* increases with an increase in servo-voltage and pulse-on time and it increases linearly with an increase in pulse on time while considerably increases with servo- voltage; (ii) *VGCS* slightly increase with an increase in wire speed; and (iii) wire tension has an insignificant effect on *VGCS*. These can be justified by the fact that higher values of spark-voltage and pulse-on time cause the occurrence of higher discharge energy of sparks which accelerate the formation of irregularly shaped craters on flank surfaces, due to which gear cutting speed increases that results in higher material removal rate. Moreover, higher pulse-on time extends the duration of transferring sparks in the inter-electrode gap which leads to the production of non-uniform deeper and wider craters results in higher material removal from flank surfaces of miniature gears. Fresh wire always comes in contact with sparks at higher wire speed which minimize sparks concentration at a certain point for a longer period of time results in improved gear cutting speed due to the absence of wire deflections and adherence of wire on the tooth flank surfaces and proper flushing of debris from the inert-electrode gap. Higher wire tension is recommended to achieve the desired dimensional accuracy by minimizing wire deflections and short-circuiting due to adherence of wire with tooth flank results in improper flushing of debris from machining zone. 

**Figure 7:**
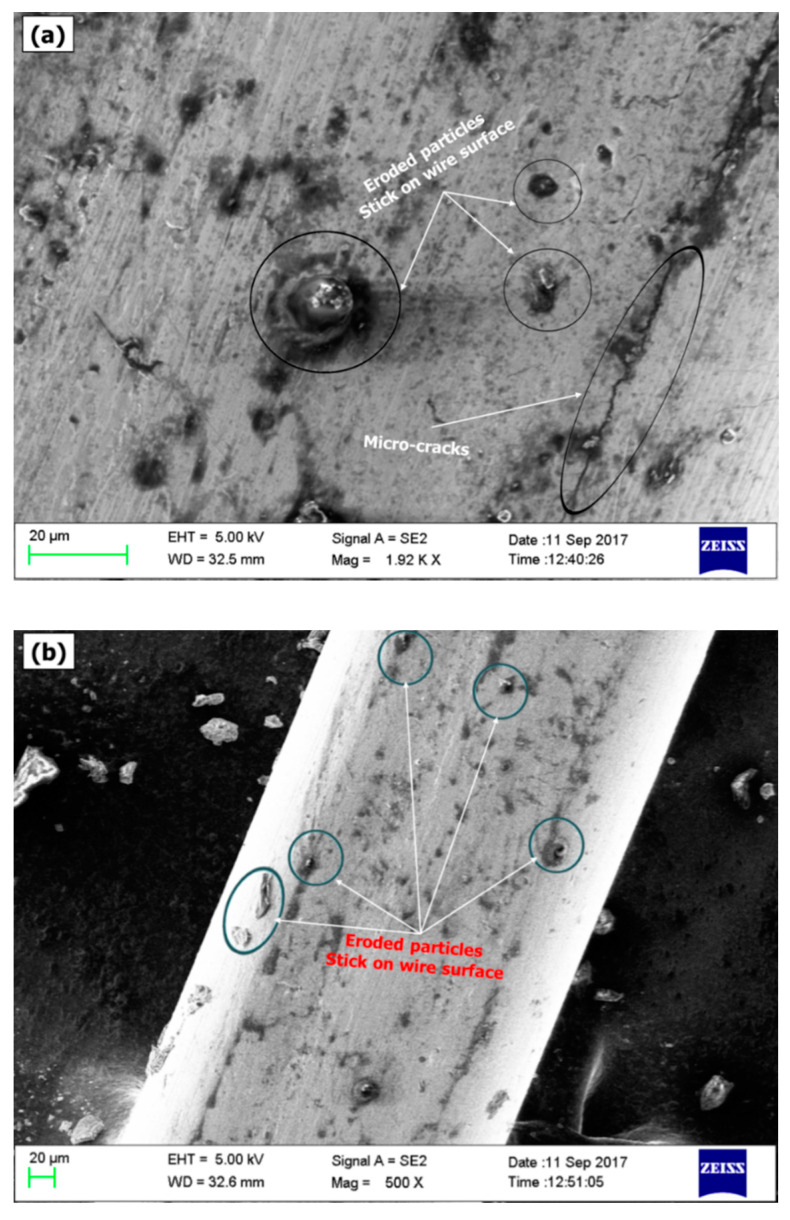
Scanning electron microscope images of the used wire after miniature gear manufacturing (**a**) presence of eroded particles and micro-cracks at higher values of pulse-on time; and (**b**) adherence of eroded particles on wire surface at lower values of wire speed.

It is evident from Figure 6a,b that *R_a_* and *R_t_* increase linearly with an increase in servo-voltage and pulse-on time. This can be explained by the fact that violent sparks are developed at higher values of these parameters which cause the formation of irregular shaped deeper and wider sparks between wire and tooth flank which leads to deterioration of the surface of tooth flank as well as wire, as shown in Figure 7a. It can be observed from Figure 6c that surface roughness parameters *R_a_* and *R_t_* slightly decrease with an increase in the speed of wire. This is due to fact that higher wire speed minimizes the sparks concentration on a particular location of the wire which decreases the wire deflections and avoids the adherence of eroded particles on wire and tooth flank surfaces. Similar trends were observed by Gupta and Jain in their investigation on WTEM of miniature gears of brass [16]. Figure 7b shows the adherence of eroded particles at lower values of wire tension and wire speed. It can be observed from Figure 6d that wire tension has insignificant effects on *R_a_* and *R_t_*. A higher value of wire tension is required to achieve desired dimensional accuracy and proper flushing of eroded particles from wire and gear tooth flank.

It is evident from Table A1 that the best finish miniature spur gear was manufactured at experiment no. 4 having WTEM parameter combination of 15 V servo-voltage; 0.9 μs pulse-on time; 5 m/min wire speed, 1620 g as wire tension, 12 A as peak current; 44.5 μs pulse-off time; 7 kg/cm^2^ as dielectric pressure, and 100% cutting speed. Whereas, maximum volumetric gear cutting speed was achieved at 20 V for servo-voltage; 1.3 μs pulse-on time; 5 m/min wire speed, 1140 g as wire tension, 12 A as peak current; 44.5 μs pulse-off time; 7 kg/cm^2^ as dielectric pressure, and 100% cutting speed. 

## 4. Validation Experiment for Surface Finish

Considering the surface finish parameters ‘*R_a_*’ and ‘*R_t_*’ more important than gear cutting speed, it was decided to perform validation experiments using parametric conditions as mentioned in experiment no. 4. Two validation experiments were performed at machining condition 15 V servo-voltage; 0.9 μs pulse-on time; 5 m/min wire speed, 1620 g as wire tension while other WTEM parameters were kept constant. Table 2 presents machine WTEM parametric combinations and the average and total height of roughness profile along with their corresponding values obtained from the experimental and validation experiments. The values of the average and total height of roughness profile reveal very good agreement between the experimental results and validation results having 1.9% and 1.5% differences. 

### 4.1. Metrological Inspection of the Miniature Gear Having Best Surface Finish

**Figure 8:**
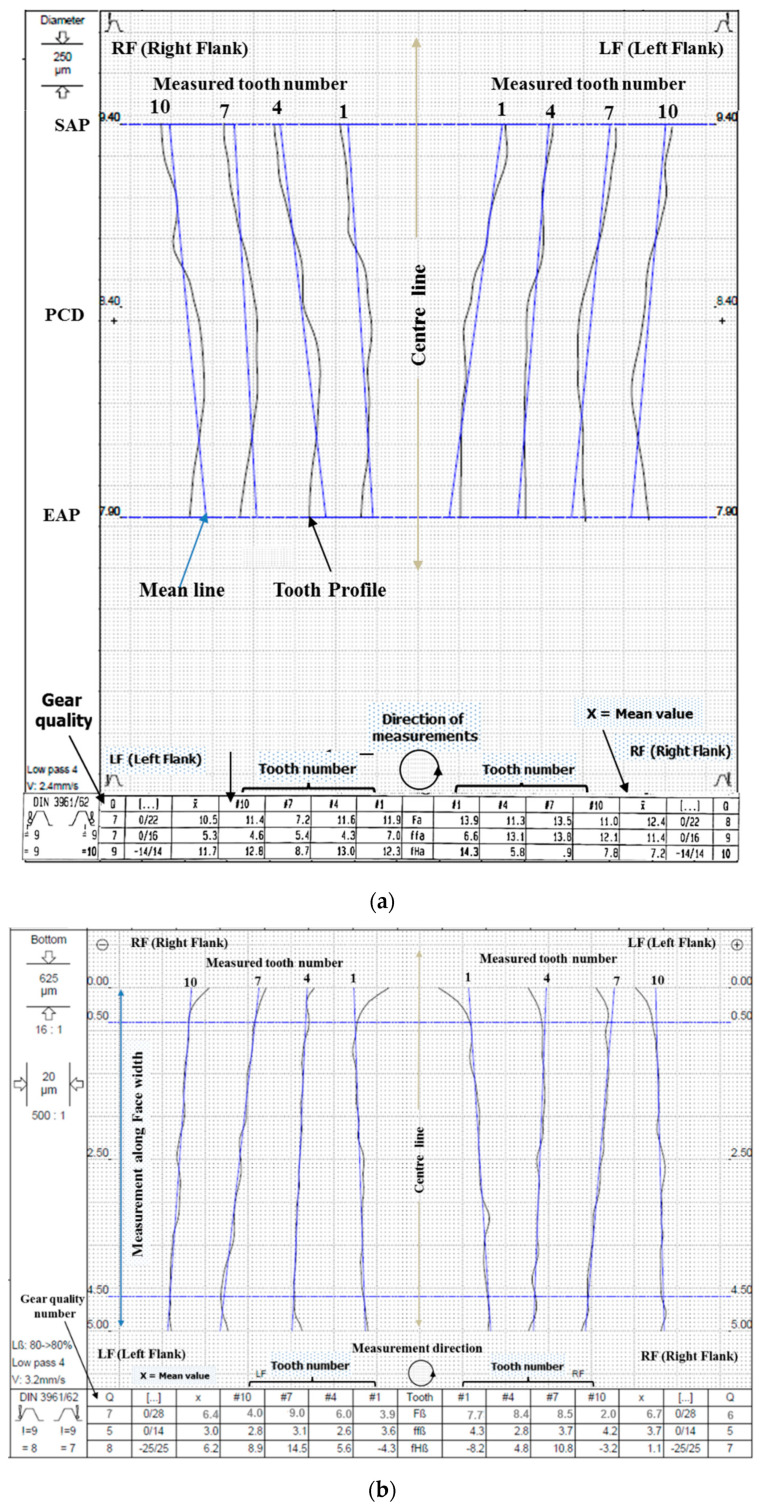

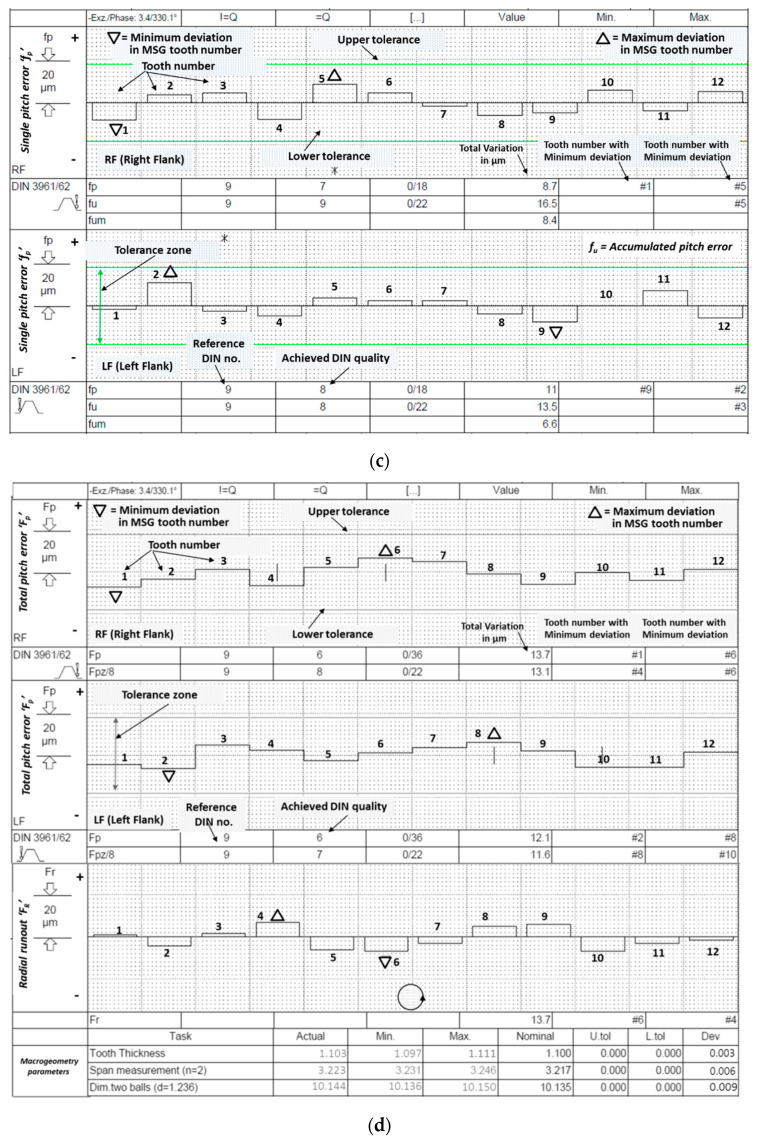
Metrological inspection report of best finish miniature gear manufactured by WTEM at optimized parameters: (**a**) total profile error ‘*F_a_*’; (**b**) helix or lead error ‘*H_ß_*’; (**c**) single pitch error ‘*f_p_*’ and adjacent pitch error ‘*f_u_*’; and (**d**) total cumulative pitch error ‘*F_p_*’ and radial runout ‘*F_r_*’.

Microgeometry measurement of WTEM machined miniature gear of aluminium was conducted to check its manufacturing quality as per DIN 3962 standard in order to estimate the performance ability of this gear. A random selection of four teeth (i.e., tooth number 1, 4, 7, and 10) for every gear was done by the metrology machine during measurement of total profile error ‘Fa’ and total lead error ‘Fß’ of both tooth flanks (i.e., right flank ‘RF’ and left flank ‘LHF’) and their mean values were calculated by machine to assign gear quality number. For pitch measurement, both flanks of all twelve teeth were inspected for single pitch error ‘fP’, accumulated pitch error ‘fu’, total pitch error ‘FP’, and radial runout error ‘Fr’. Figure 8a–d presents the microgeometry measurement results as 11.45 μm total profile error, 6.55 μm total lead error, 9.85 μm single pitch error; 15.0 μm adjacent pitch error; 12.9 µm total pitch error; and 13.7 μm radial runout. Figure 8d shows deviations in tooth thickness and span as 3 μm and 6 μm respectively. The results of this metrological inspection are summarized in Table 3. Overall, an average DIN quality assigned to this gear is DIN 7. In similar research on WTEM of miniature gears of brass, average roughness *R_a_* value up to 1 μm and maximum roughness around 6 μm was obtained [13]. Another interesting past work on WTEM of miniature gears of stainless steel reported *R_a_* value less than 1 μm and *R_max_* value 6 μm [17]. Based on the deviation in microgeometry, manufacturing quality of DIN 7 was assigned to miniature gears in both of the past research works. Since the present work is a preliminary investigation on WTEM of miniature gears on aluminium, some future attempts are required to further optimize the WTEM parameters to obtain better or improved surface roughness and microgeometry characteristics.

**Figure 9:**
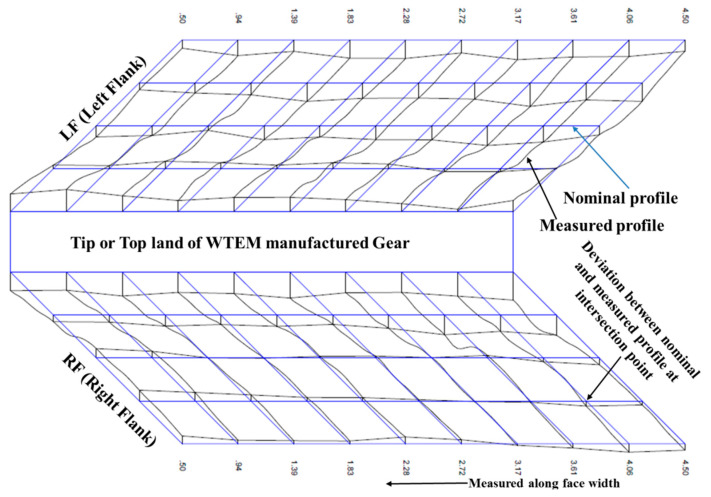
Topography of miniature gear manufactured using optimized machining parameters.

Figure 9 represents the three-dimensional view of both flanks of any random tooth. The topography inspection of miniature gear reveals very little variation between theoretical surface (i.e., blue line) and actual surface (i.e., black line) that is due to uniform distribution of regular-shaped and undipped craters on flank surface during WTEM. This ensures the good functional performance of the lightweight aluminium miniature gear developed by the WTEM process in this work.

### 4.2. Microscopic Examination of the Best Finish Miniature Gear

**Figure 10:**
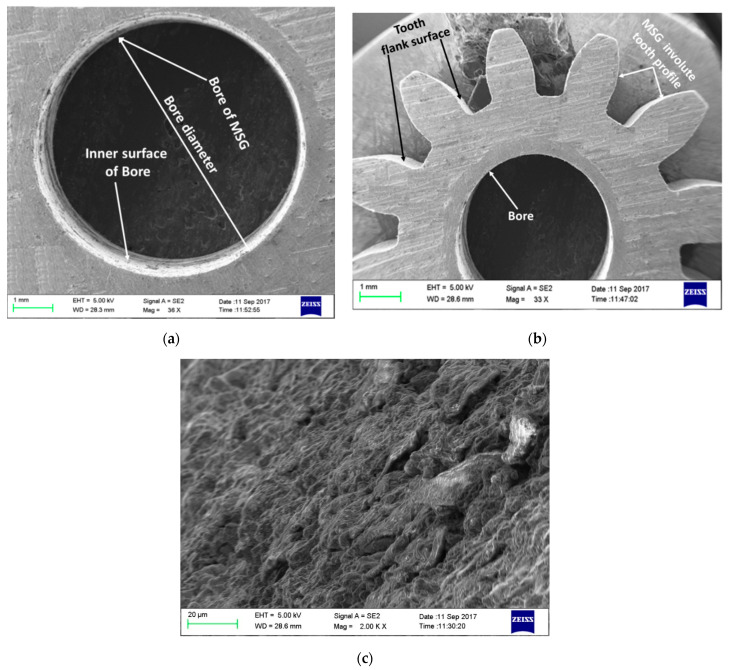
Scanning electron microscope micrographs of WTEM manufactured miniature gear showing (**a**) bore diameter free from burr and sharp edges on both top and bottom side; (**b**) uniform and accurate tooth profile and burr-free edge definition; and (**c**) microstructure of tooth flank surface.

Figure 10 illustrates micrographs obtained from scanning electron microscopy of the miniature gear fabricated by WTEM at the validation experiment. These high-resolution 3D images reveal that bore and tooth of miniature gear manufactured by WTEM have a uniform and accurate profile, free from burr and sharp edges on both end faces with no undercut at the root (Figure 10a,b). The variation in the profile of bore (i.e., from its centre) and gear teeth (i.e., from its nominal profile) throughout the face width is responsible for noise during operation and significantly affect the performance and service life of the gear. Figure 10c shows that tooth flanks of gear have a uniform surface pattern free from cracks, machining streaks, and accumulation of worn particles. Uniform surface pattern of WTEM manufactured gear tooth flank indicates the uniformly distributed craters of similar depth and size. 

## 5. Conclusions

This paper has reported a study focused on the manufacturing of miniature size gears with bore and hub and effects of considered variable parameters of wire-assisted thermoelectric erosion machining (WTEM) process on gear quality. The paper has comprehensively described the development of lightweight aluminium spur gear of miniature size by wire-assisted WTEM process. The results of this study revealed that manufactured gears are suitable for precision applications. Referring to the original aims and objectives the following conclusions can be drawn from the present work:

- Explored the potential of the WTEM process by successfully manufacturing lightweight (<0.8 g) aluminium miniature spur gears with bore and hub.
- Identified servo-voltage and pulse-on time as the most significant parameters which affect the surface finish of miniature size spur gear and productivity of the WTEM process.
- Achieved surface finish with an average and total height of roughness profile values up to 1.58 μm and 9.37 µm respectively.
- Achieved volumetric gear cutting speed up to 12.83 mm^3^/min.
- Best finish gear revealed gear quality up to DIN 7 standard number.
- Microscopic investigation revealed accurate and uniform tooth profiles free from burr and sharp edge definition. Moreover, tooth flank surfaces were found free from cracks and contamination.

A detailed explanation of the development of gears with bore and hub by WTEM process and the results of the present study will be very favourable for the researchers and industry users in the field of WTEM for manufacturing high-quality lightweight miniature gears with bore and hub. 

## Figures and Tables

**Table 1:** Values of the constant parameters, levels, and corresponding values of the variable parameters of WTEM used in the experiments.

Variable Parameters				Constant Parameters
WTEM Parameters (Units)	Levels			
	A	B	C	
Pulse-on time ‘*T_on_*’ (µs)	0.9	1.1	1.3	Peak current (*I_P_*): 12 A; Cutting speed (*C_S_*): 100 %; Pulse-off time ‚*T_off_*: 44.5 μs; Wire material: soft plain brass (Tensile strength: 470–510 N/mm^2^); Wire diameter: 0.25 mm; Dielectric: deionized water; and Dielectric pressure (*W_P_*): 7 kg/cm^2^
Servo-voltage ‘*S_v_*’ (Volts)	10	15	20	
Wire speed ‘*W_S_*’ (m/min)	3	5	7	
Wire tension ‘*W_T_*’ (g)	1140	1380	1620	
**Composition of gear material (% weight):** 0.4% Si; 2.1% Mg; 0.15% Mn; 1.2% Cu; 0.18% Cr; 0.2% Si; 5.3% Zn; 0.1% Ti, and balance Aluminium				
**Detailed gear specifications*:*** Outside diameter: 9.8 mm; Bore diameter: 3.0 mm; Hub diameter: 6.0 mm; Pitch circle diameter: 8.4 mm; module: 0.7 mm; Root diameter: 6.65 mm; Face width: 5.0 mm; Hub length: 5.0 mm; Number of tooth: 12; Gear material: Aluminium alloy (7075)				
**Specification of WTEM Machine** Resolution: 0.0005 mm; Table size: 440 × 650 mm; Taper angle: ±300 Max. workpiece height : 200 mm; Max. workpiece weight: 300 kg Max. jog speed: 900 mm/min; Max. wire spool capacity/dia: 6 kg/ 0.25 mm standard, 0.15, and 0.2 optional				

**Table 2:** Results of validation experiments for the best finish miniature gear manufactured by WTEM.

Values of Variable WTEM Parameters				Surface Roughness Parameters (μm)						Difference (% Error)	
				*R_a_*			*R_t_*		Avg.		
*S_V_* (Volts)	*T_on_* (µs)	*W_S_* (m/min)	*W_T_* (g)	*R1*	*R2*	Avg.	*R1*	*R2*		*R_a_*	*R_t_*
15	0.9	5	1620	-	-	-	-	-	-	-	-
Validation				1.54	1.56	1.55	9.61	8.85	9.23	0.02 (1.9)	0.14 (1.5)
Experimental				1.58			9.37				

**Table 3:** Results of metrological inspections of the best finish miniature spur gears.

Criteria for Evaluation	Best Quality Meso Helical Gear of Al			
	Value			Quality
Microgeometry Parameters	RHF	LHF	Average	
Total profile error ‘*F_a_*’ (µm)	12.4	10.5	11.45	*DIN* 7
Total lead error ‘*F_ß_*’ (µm)	6.4	6.7	6.55	*DIN* 7
Single pitch error ‘*f_p_*’ (µm)	11.0	8.7	9.85	*DIN* 6
Successive pitch error ‘*f_u_*’ (µm)	13.5	16.5	15.00	*DIN 8*
Total pitch error ‘*F_p_*’ (µm)	12.1	13.7	12. 9	*DIN* 6
Radial runout ‘*F_r_*’ (µm)	13.7			*DIN 8*
**Macrogeometry parameters**	**Deviation**			
Tooth thickness deviation (µm)	3			
Span deviation (µm)	6			

## Appendix A

**Table A1:** Values of variable WTEM parameters for nine experimental runs and corresponding average values (i.e., replicates *R1 and R2*) of considered responses for miniature gear fabrication.

Exp. Runs	Variable WTEM Parameters				Weight (g)					Mat. Lost (W_L_)	M/C Time ‘t’ (min.)	Responses		
	*S_V_* (Volts)	*T_on_* (μs)	*W_S_* (m/min)	*W_T_* (g)	Gear Blank (W = W_B_ + W_A_)		Gear (W_g_)	Bore Bar (W_b_)	W_A_ + W_g_ + W_b_			VGCS (mm^3^/min)	Surface Roughness (μm)	
					Before	After							Avg. Values *R_a_* (*R1* + *R2*)	Avg. Values *R_t_* (*R1* + *R2*)
1	10	0.9	3	1140	1.84	0.69	0.74	0.11	1.54	0.31	17:30	6.30	1.65	9.48
2	10	1.1	5	1380	1.98	0.72	0.77	0.11	1.60	0.38	14:36	9.26	1.68	9.8
3	10	1.3	7	1620	1.87	0.68	0.76	0.12	1.56	0.31	09:21	12.21	1.70	10.2
4	15	0.9	5	1620	1.92	0.73	0.75	0.1	1.58	0.34	16:40	7.26	1.58	9.37
5	15	1.1	7	1140	1.95	0.73	0.76	0.11	1.60	0.35	12:42	9.81	1.71	11.2
6	15	1.3	3	1380	1.92	0.69	0.79	0.11	1.59	0.33	10:15	11.47	1.81	12
7	20	0.9	7	1380	1.92	0.7	0.76	0.1	1.56	0.36	15:30	8.28	1.62	10.12
8	20	1.1	3	1620	1.91	0.73	0.72	0.1	1.55	0.36	13:17	9.65	1.79	11.4
9	20	1.3	5	1140	1.96	0.74	0.76	0.11	1.61	0.35	11:30	12.83	1.83	12.4

**Table A2:** Results of regression analysis and ANOVA for all responses considered in this work.

**Regression Analysis: VGCS Versus *S_V_*, *T_on_*, *W_S_*, and *W_T_***					
**Predictor**	**Coefficient**	**SE Coefficient**	***T*** **Test**	***p*** **Value**	
Constant	−6.6406	0.8449	−7.86	**0.001**	
Servo-voltage ‘*S_V_*’	0.09967	0.01898	5.25	**0.006**	
Pulse-on time ‘*T_on_*’	12.2250	0.4746	25.76	**0.001**	
Wire speed ‘*W_S_*’	0.24000	0.04746	5.06	**0.007**	
Wire tension ‘*W_T_*’	0.0001250	0.0003955	0.32	0.768	
**Standard Error (S)** = 0.232516 **R-Square** = 99.4% **Adjusted R-Square** = 98.9%					
**Analysis of Variance (ANOVA)**					
**Source**	**Degree of Freedom**	**Sum of Square**	**Mean Square**	**F Test**	***p*** **Value**
Regression	4	38.7460	9.6865	179.17	**0.000**
Residual Error	4	0.2163	0.0541		
Total	8	38.9622			
**Regression Analysis: *R_a_* Versus *S_V_*, *T_on_*, *W_S_*, and *W_T_***					
Constant	1.36028	0.09125	14.91	**0.001**	
Servo-voltage ‘*S_V_*’	0.007000	0.002050	3.41	**0.027**	
Pulse-on time ‘*T_on_*’	0.40833	0.05126	7.97	**0.001**	
Wire speed ‘*W_S_*’	−0.018333	0.005126	−3.58	**0.023**	
Wire tension ‘*W_T_*’	−0.00008333	0.00004271	−1.95	0.123	
**Standard Error (S)** = 0.0251109 **R-Square** = 95.8% **Adjusted R-Square** = 91.6%					
**Analysis of Variance (ANOVA)**					
**Source**	**Degree of Freedom**	**Sum of Square**	**Mean Square**	**F Test**	***p*** **Value**
Regression	4	0.057833	0.014458	22.93	**0.005**
Residual Error	4	0.002522	0.000631		
Total	8	0.060356			
**Regression Analysis: *R_t_* Versus *S_V_*, *T_on_*, *W_S_***, and ***W_T_***					
Constant	5.871	1.604	5.52	**0.005**	
Servo-voltage ‘*S_V_*’	0.14800	0.02391	6.19	0.003	
Pulse-on time ‘*T_on_*’	4.6917	0.5977	7.85	0.001	
Wire speed ‘*W_S_*’	−0.11333	0.05977	−1.90	0.131	
Wire tension ‘*W_T_*’	−0.0014653	0.0004980	−2.94	0.042	
**Standard Error (S)** = 0.292788 **R-Square** = 96.6% **Adjusted R-Square** = 93.1%					
**Analysis of Variance**					
**Source**	**Degree of Freedom**	**Sum of Square**	**Mean Square**	**F Test**	***p*** **Value**
Regression	4	9.6187	2.4047	28.05	**0.003**
Residual Error	4	0.3429	0.0857		
Total	8	9.9616			
